# Electrochemical Behavior of Nanoporous Gold/Polypyrrole Supercapacitor under Deformation

**DOI:** 10.3390/nano12132149

**Published:** 2022-06-22

**Authors:** Jie Li, Liang-Yu Li, Peng Jia, Ilya V. Okulov

**Affiliations:** 1 Materials Mechanics, Institute of Materials Research, Helmholtz-Zentrum Hereon, Max-Planck-Str. 1, 21502 Geesthacht, Germany; 2 Department of Mechanical and Aerospace Engineering, Hong Kong University of Science and Technology, Clear Water Bay, Hong Kong, China; liliangyu@ust.hk; 3 State Key Laboratory of Biobased Material and Green Papermaking, Key Laboratory of Pulp and Paper Science & Technology of Ministry of Education/Shandong Province, Faculty of Light Industry, Qilu University of Technology (Shandong Academy of Sciences), Jinan 250353, China; skl_jiapeng@163.com; 4 Leibniz Institute for Materials Engineering-IWT, Badgasteiner Str. 3, 28359 Bremen, Germany; 5 Faculty of Production Engineering, University of Bremen, Badgasteiner Str. 1, 28359 Bremen, Germany

**Keywords:** dealloying, nanoporous gold, polypyrrole, electrochemical behavior, deformation

## Abstract

Due to the high demand of wearable electronics, flexible supercapacitors have been extensively developed in recent years. Yet, the effect of deformation in the interior electrode material suffered in practical applications on the performance received less attention. Here, we study the electrochemical behavior of macroscopic nanoporous gold/polypyrrole (NPG/PPy) in situ under compression deformation. Dealloying-driven NPG, a network constructed by bi-continuous nano-scaled ligaments and pores, can serve as a compression-tolerant substrate for PPy supercapacitor material. The electrochemical capacitance of NPG/PPy subjected to compression deformation is revealed to decrease at the scan rates and discharge current densities applied in this work. At the same time, the charge transfer resistance of NPG/PPy is found to increase. This electrochemical behavior is due to the locally reduced mass transport of electrolyte caused by the formation of new connections between the neighboring ligaments under the application of compression loads. The fundamental understanding of the effect of deformation on the performance of energy storage materials revealed in this study paves the way for their practical application in wearable devices.

## 1. Introduction

In order to meet the growing demand for green energy, high-performance energy-storage and conversion devices, such as batteries, electrochemical supercapacitors (ESCs), and fuel cells have been extensively explored [1]. Among them, ESCs are expected to bridge the gap between conventional electrolytic capacitors featuring high-power density but low-energy density and batteries characterized by high-energy density yet low-power density and short lifetime. According to the charge storage mechanisms, the ESCs are sorted into electrochemical double-layer capacitors (EDLCs) where energy is stored via charge carriers adsorption/desorption in the double layer at electrode/electrolyte interface [2,3] and pseudocapacitors, which effectuate charge storage by means of redox reactions at the surface [4,5]. Thus, pseudocapacitors are expected to possess higher energy density and larger capacitance than EDLCs.

Transition-metal oxides/hydroxides, such as RuO${}_{2}$ [6], MnO${}_{2}$ [7], and Ni(OH)${}_{2}$ [8], and conductive polymers (CPs), such as polyaniline (PANI) [5,9] and polypyrrole (PPy) [5,10] are most commonly used as electrode materials for pseudocapacitors. Amongst diverse pseudocapacitor materials, PPy has been extensively investigated owning to the facile synthesis, low cost, environmental friendliness, and high capacitance [11]. However, the low electric conductivity and the structure failure induced by the consecutive expansion and contraction during redox processes limits the capacitance of PPy-based pseudocapacitors. The performance of PPy-based devices can be optimized by incorporating PPy on conductive substrates, for instance, carbonaceous materials [12,13,14] and metallic foams [15]. Beneficial to the excellent conductivity and mechanical robustness, chemical and/or electrochemical corrosion driven nanoporous gold (NPG, note the term “nano” is meaningless from the standpoint of IUPAC (International Union of Pure and Applied Chemistry) nomenclature, where “micro”, “meso”, and “macro” refers to materials with pores size of <2 nm, 2–50 nm, and >50 nm, respectively. In the field of dealloying, the prefix “nano” refers to ligaments and/or pores size ranging from a few to hundreds of nm.) has been one of the most bright substrate candidates [16,17,18,19,20,21]. NPG is a bi-continuous network constructed by ligaments and pores, both of which can be tuned from several to hundreds of nm, giving rise to large specific surface area, providing numerous sites for the deposition of foreign active materials [16,22,23,24,25]. In addition, the porous structure can evidently eliminate structure damage caused by the volume change during electrochemical processes [26] while persevering efficient electrolyte transport.

In the past decade, novel nanoporous gold/polypyrrole (NPG/PPy) hybrid electrodes for supercapacitors have been fabricated [27,28,29,30,31]. The PPy thickness was elaborately selected to keep open porous structure. The open porous NPG/PPy structure enables the pathway for electrolyte diffusion and provides the space to buffer the volume change during redox processes. In these works, NPG/PPy hybrid materials supercapacitors exhibit enhanced performance over individual components (i.e., NPG and PPy). Moreover, the NPG/PPy hybrid electrodes are robust enough to be integrated into flexible and wearable devices for applications. Nevertheless, one should note that the supercapacitors will undergo various distortions, such as stretching, compressing, bending, twisting, and folding in different situations. As a result, the microstructure will be deformed and the electrochemical behavior will be affected. It has been documented that under compression the collapse of the Graphene sheets and the accompanying reduction of surface area will lead to a lower power density [32]. Yet, for the compressed single-walled carbon nanotube supercapacitor, the increased surface wettability of the electrolyte will improve the specific capacitance [33]. Although nonporous metal-based supercapacitors have been developed, their electrochemical performance under application of mechanical stresses and deformation has not been investigated.

In this work, we report on the effect of compressive deformations on the electrochemical behavior of the NPG/PPy hybrid electrode. The mm-scaled cylindrical specimens were explored for ease of compression. The new connections are formed between the neighboring ligaments under compression. The impacts of the microstructure variation on the mass transfer and the electrochemical capacitance were studied.

## 2. Materials and Methods

### 2.1. Preparation of NPG/PPy Hybrid Materials

NPG/PPy hybrid materials were fabricated following the procedures in Ref. [18]. Au${}_{25}$Ag${}_{75}$ alloy ingots, produced by arc-melting Au and Ag (99.99 wt.%, Alfa Aesar GmbH & Co KG, Germany) under the protection of argon (Ar) atmosphere, were homogenized at 850 ${}^{\circ }$C for 120 h under vacuum. In the next step, the ingots were drawn into wires with a diameter of ∼1.2 mm and were then cut by a diamond wire saw (Model 3032, WELL Diamond Wire Saws SA, Germany) in the form of a cylinder with a height of ∼1.8 mm. The residual stress introduced during drawing and cutting was realized by annealing the cylinders at 800 ${}^{\circ }$C for 2 h in Ar (IRF 10, behr Labor Technik Gmbh, Germany). NPG specimens were made from electrochemical dealloying Au${}_{25}$Ag${}_{75}$ cylinders in 1 M HClO${}_{4}$ (60%, AppliChem GmbH, Germany) at room temperature (RT) in a three-electrode electrochemical cell with Ag wire as counter electrode (CE) and Ag/AgCl as pseudo reference electrode (RE, 0.515 V vs. standard hydrogen electrode, SHE). A constant potential of 0.75 V (vs. pseudo Ag/AgCl) was applied to deplete Ag out of Au${}_{25}$Ag${}_{75}$ cylinders until the current dropped below 10 µA. Ag content was further reduced below 2 at.% (as seen in Figure S1 in the Supplementary Materials) through 15 cycle potential sweeps between −0.5 and 1.0 V (vs. pseudo Ag/AgCl) at a scan rate of 5 mV s${}^{-1}$ in 1 M HClO${}_{4}$ (70%, Suprapur, Merck & Co., USA). The dealloyed samples were rinsed with ultrapure water (18.2 MΩ·cm, Siemens Ultra Clear TWF UV TM) and ethanol at least three times to remove residual acid and metal ions. NPG microstructure was coarsened by annealing at 300 ${}^{\circ }$C for 30 min in the air (IRF 10, behr Labor Technik Gmbh, Germany). NPG/PPy hybrid materials were fabricated by electropolymerizing pyrrole monomers on NPG. Prior to electropolymerization, pyrrole monomer liquid (98%, Sigma-Aldrich Chemie GmbH, Germany) was purified by flowing through Si particles (0.063–0.200 mm particle size, Sigma-Aldrich Chemie GmbH, Germany). Electropolymerization was conducted in a one-chamber electrochemical cell under the protection of Ar at RT. Annealed NPG, Pt mesh, and Ag/AgCl (filled with saturated KCl, Metrohm AG, Switzerland), respectively, working as WE, CE, and RE were placed in Acetonitrile solvent containing 0.3 M LiClO${}_{4}$, 0.3 M purified pyrrole, and 2 vol.% H_2_O. In this work, pulse electropolymerization was employed to obtain homogeneous PPy coating throughout the mm-scaled specimens [10]. In one typical electropolymerization cycle, a 0.8 V potential was applied for 1 s then the potential was switched off for 60 s to allow the pyrrole monomers to diffuse into NPG inner pores for the subsequent electropolymerization cycle. By adjusting pulse cycles, NPG/PPy hybrid materials with PPy coating thickness ranging from 0 to 30 nm were fabricated. The NPG/PPy hybrid materials were rinsed with ultrapure water and ethanol and dried in a fume hood.

### 2.2. Characterizations and Instruments

The microstructure and composition of NPG and NPG/PPy specimens were characterized by a scanning electron microscope (SEM, Zeiss Supra VP55) coupled with energy dispersive spectroscopy (EDS). All electrochemical experiments were carried out with an Autolab PGSTAT302N potentiostat (Metrohm).

### 2.3. Electrochemical Measurements

The electrochemical properties of NPG/PPy hybrid materials and NPG were inspected in a three-electrode system in situ under uniaxial compression. The compressive deformations were carried out with a mechanical device (Zwick/Roell Z010) and all samples were successively deformed to 0%, 2.5%, 7.5%, and 15%, respectively. Under each specific deformation, cyclic voltammograms (CV) and galvanostatic charge-discharge (GCD) curves were recorded in a potential range of 0∼0.8 V at various scan rates and current densities. The electrochemical impedance spectroscopy (EIS) was performed at frequencies from 0.01 to 100 kHz with a sinusoidal potential amplitude of 10 mV, which superimposed to a DC formal potential of 0.5 V and 0.4 V, the middle potential in CV, for bare NPG and NPG/PPy hybrid materials, respectively. All electrochemical measurements were performed in the 0.1 M HClO${}_{4}$ (70%, Suprapur, Merck) at RT using the explored specimen, pseudo Ag/AgCl, and carbon cloth as WE, RE, and CE, respectively.

In this work, all potentials were normalized to SHE unless otherwise stated.

## 3. Results

### 3.1. Microstructure

**Figure 1:**
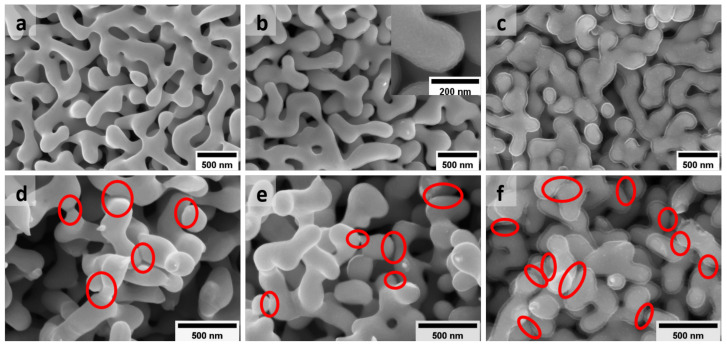
SEM images showing the typical microstructure of as-prepared (**a**) bare NPG (with mean ligament size of L=150±40 nm) and NPG/PPy hybrid materials with (**b**) the thinnest ($t_{\mathrm{PPy}}=11\pm 2$ nm) and (**c**) the thickest ($t_{\mathrm{PPy}}=29\pm 3$ nm) PPy layer. The inset in (**b**) illustrates one representative NPG/PPy strut at a higher mignification where PPy coating is clearly seen on NPG ligament. (**d**–**f**) Microstructure of NPG, NPG/PPy(10 nm), and NPG/PPy(30 nm) after deformation of 15%, respectively. The red ellipses indicate the “bridging” of neighboring ligaments.

Figure 1 illustrates the microstructure of as-prepared bare NPG and NPG/PPy hybrid materials. NPG is characterized by a 3D bicontinuous network constructed by nano-scaled ligaments and pores, as shown in Figure 1a. The mean ligament size of NPG is L=150±40 nm (based on averaging over 30 measurements). It well matches the characteristic size (156 nm) predicted by $g/\alpha_{V}$ [34], with the volume specific surface area of $\alpha_{V}$∼ 80 cm${}^{2}$/mm${}^{3}$, which was obtained based on the capacitance ratio method (Figure S2) [35,36] and the geometry constant of *g* = 4. Among the ligaments are the pores that have a comparable feature size with the surrounding ligaments, providing pathways for electrolyte flux for the electrochemical process. Figure 1b depicts NPG ligaments coated by a uniform PPy layer. The inset of Figure 1b displays one representative strut at a higher magnification clearly illustrating the thin PPy layer with a thickness of $t_{\mathrm{PPy}}=11\pm 2$ nm (simplified as NPG/PPy(10 nm) herein and after), which corresponds to an electrodeposition charge density of $q_{A}$ = 3.3 mC/cm${}^{2}$ [18]. The small thickness around 10 nm was intentionally chosen to remain the ligament and pore sizes as much as similar to those of bare NPG. On the other hand, a PPy layer of $t_{\mathrm{PPy}}=29\pm 3$ nm (NPG/PPy(30 nm), $q_{A}$ = 10 mC/cm${}^{2}$) was elaborately electrodeposited, as shown in Figure 1c. The pores between the enlarged ligaments in NPG/PPy(30 nm) are much smaller than those in NPG/PPy(10 nm), however, they still remain open.

NPG and NPG/PPy specimens are intrinsically strong to sustain a large load of 40 MPa (Figure S3). The microstructure characteristic, e.g., porosity, can be elaborately adjusted simply by deforming, predestining them for model materials to study the electrochemical behavior under deformation. Figure 1d–f show the microstructure of bare NPG, NPG/PPy(10 nm), and NPG/PPy(30 nm) after deformation of 15%, respectively. It can be seen that after deformation, new connections (alternatively called “bridging” [26]) are formed between neighboring ligaments, as marked by the red ellipses in the figures. As seen from SEM micrographs, more connections are formed in the deformed NPG/PPy(30 nm) in comparison with bare NPG and NPG/PPy(10 nm), which illustrates a fairly similar amount of new connections after compression.

### 3.2. Cyclic Voltammetry

**Figure 2:**
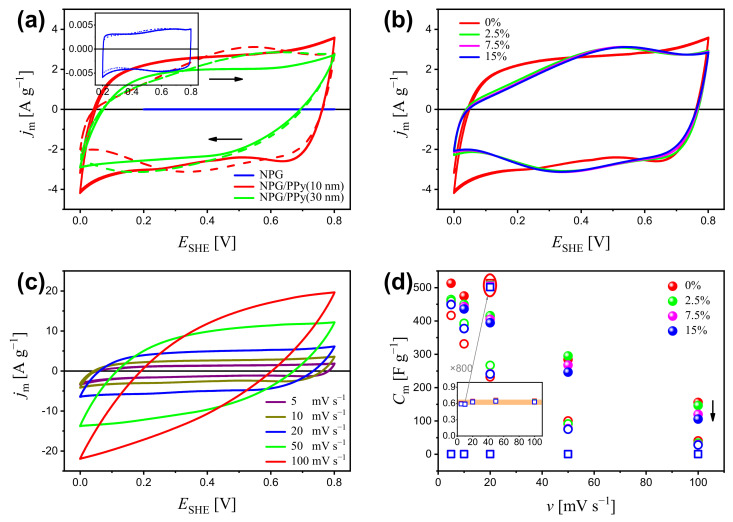
(**a**) Cyclic voltammogram (CV) of NPG, NPG/PPy(10 nm), and NPG/PPy(30 nm) under deformations of 0% (solid lines) and 15% (dashed lines) at a scan rate of v=10 mV s${}^{-1}$. Inset outlines the CV curves of NPG at a higher magnification. (**b**) CV curves of NPG/PPy(10 nm) under various deformations of 0∼15% at v=10 mV s${}^{-1}$. (**c**) CV curves of non-deformed NPG/PPy(10 nm) at *v* = 5∼100 mV s${}^{-1}$. (**d**) Specific capacitance, $C_{m}$, of NPG, NPG/PPy(10 nm), and NPG/PPy(30 nm) under various deformations versus scan rates based on the CV curves. Inset shows the $C_{m}$ of NPG at a higher magnification. The $C_{m}$ values of NPG obtained at 20 mV s${}^{-1}$ are magnified by 800 times, as marked by the gray arrow and red ellipse. The black arrow indicates the decrease of $C_{m}$. The open squares, closed symbols, and open circles, respectively, represent NPG, NPG/PPy(10 nm), and NPG/PPy(30 nm), and they use the same color conventions to indicate the deformation.

Figure 2 shows the cyclic voltammogram (CV) of NPG, NPG/PPy(10 nm), and NPG/PPy(30 nm) under different deformations as well as their corresponding specific capacitance, $C_{m}$. In order to avoid the hydrogen evolution (Figure S4 in Supplementary Materials) and the over-oxidation of PPy [37,38], NPG/PPy hybrid materials were explored in a potential of 0∼0.8 V while bare NPG was explored in a potential range of 0.2∼0.8 V. Non-deformed NPG/PPy hybrid materials and bare NPG display quasi-rectangular current response in the investigated potential range, as shown in Figure 2a. The magnitude of current density, $j_{m}$, follows NPG/PPy(10 nm) > NPG/PPy(30 nm) ≫ NPG. Under a deformation of 15%, NPG shows an identical current response to that obtained under zero deformation. Yet, distorted current profiles are revealed in the cases of 15%-deformed NPG/PPy hybrid materials, as shown in Figure 2a. Figure 2b presents the CV curves of NPG/PPy(10 nm) under the studied deformations. All current responses (at engineering strains of 2.5%, 7.5%, and 15%) feature a malformed rectangular shape and are comparable in magnitudes.

The electrochemical behavior was examined at various scan rates, *v*. The CV curves of non-deformed NPG/PPy(10 nm) are representatively displayed in Figure 2c. It is seen that the $j_{m}$ increases with *v*, and the quasi-rectangular profile is well maintained until 20 mV s${}^{-1}$, beyond which, the CV curves deviate from the rectangular shape. The specific capacitance based on the CV results is plotted in Figure 2d. As seen in the figure, at all studied scan rates, NPG/PPy(10 nm) shows higher specific capacitance than NPG/PPy(30 nm), whereas, NPG exhibits the lowest ones. Specifically, the $C_{m}$ value decreases from ∼500 F g${}^{-1}$ to ∼150 F g${}^{-1}$ for NPG/PPy(10 nm) and from ∼400 F g${}^{-1}$ to ∼30 F g${}^{-1}$ for NPG/PPy(30 nm) with increasing scan rate from 5 mV s${}^{-1}$ to 100 mV s${}^{-1}$. The $C_{m}$ value of NPG is independent of the scan rates at a magnitude of ∼0.6 F g${}^{-1}$ (Figure 2d). It has also been observed that $C_{m}$ decreases with deformation. In the case of NPG/PPy(10 nm), $C_{m}$ values at various deformations are well distinguished at all studied scan rates, especially at v>50 mV s${}^{-1}$ (as indicated by the black arrow). Moreover, $C_{m}$ values of NPG are also impacted by deformation, as marked by the red ellipse.

### 3.3. Galvanostatic Charge-Discharge

**Figure 3:**
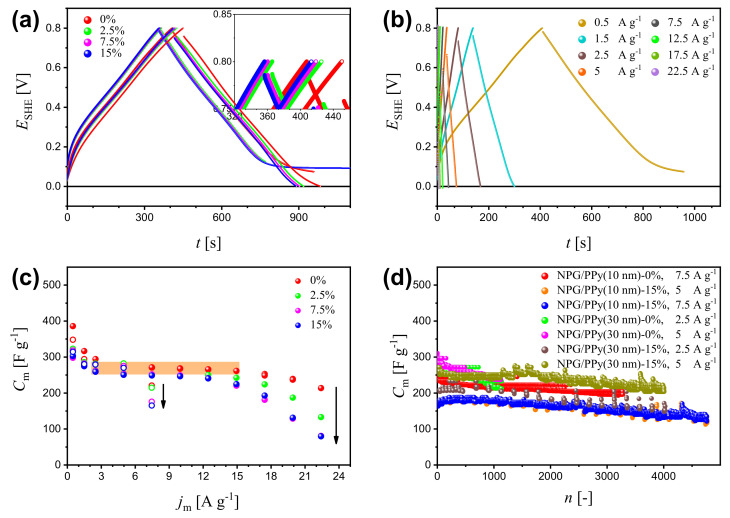
(**a**) Galvanostatic charge-discharge (GCD) curves of NPG/PPy(10 nm) and NPG/PPy(30 nm) at a current density of $j_{m}=0.5$ A g${}^{-1}$ under various deformations. Closed and open symbols represent NPG/PPy(10 nm) and NPG/PPy(30 nm), respectively. The colors indicate the deformation; the inset at a higher magnification distinguish the curves. (**b**) GCD curves of NPG/PPy(10 nm) at $j_{m}$ = 0.5∼22.5 A g${}^{-1}$ under zero deformation. (**c**) Specific capacitance, $C_{m}$, as a function of $j_{m}$ based on the GCD results of NPG/PPy(10 nm) and NPG/PPy(30 nm). Symbol and color conventions follows those in (**a**). (**d**) Cycling performance of NPG/PPy(10 nm) and NPG/PPy(30 nm) under deformations of 0% and 15%. The current densities are labeled in the figure.

Galvanostatic charge-discharge (GCD) is an effective way to evaluate the specific capacitance of active materials. Figure 3a displays the charging-discharging curves of NPG/PPy(10 nm) and NPG/PPy(30 nm) at current density $j_{m}=0.5$ A g${}^{-1}$ under various deformations (please refer to Figure S5 in Supplementary Materials for GCD results of bare NPG). NPG/PPy(30 nm) exhibited linear and symmetric GCD curves, whereas, NPG/PPy(10 nm) showed plateaus at the end of the discharging process. Moreover, larger potential drops were observed in NPG/PPy(30 nm) in comparison with those in NPG/PPy(10 nm). However, the magnitude of the potential drop of individual specimen was unvaried, even at larger deformations. The charging and discharging (specifically of the linear part) time of the non-deformed specimens were longer than those obtained under deformations, where the GCD curves nearly overlap. Figure 3b displays the GCD curves of non-deformed NPG/PPy(10 nm) at various current density of $j_{m}$ = 0.5∼22.5 A g${}^{-1}$. The plateau in the discharging curve at $j_{m}=0.5$ A g${}^{-1}$ was absent at larger current densities. However, pronounced potential drops were recognized as the current density increased. Specific capacitance values were evaluated from the GCD curves and displayed in Figure 3c. It is evident that $C_{m}$ values of both NPG/PPy hybrid materials decline with deformation at all studied current densities, as indicated by the black arrows. $C_{m}$ of both NPG/PPy hybrid materials decreases with $j_{m}$ until $j_{m}=2.5$ A g${}^{-1}$. At this $j_{m}$ value $C_{m}$ stabilized at about 260 F g${}^{-1}$, as marked by the orange rectangular in Figure 3c. In the case of non-deformed NPG/PPy(10 nm), $C_{m}$ remains invariant up to 15 A g${}^{-1}$, beyond which, $C_{m}$ gradually decreased. Yet, when NPG/PPy(10 nm) was deformed, a steady decrease of $C_{m}$ was initiated at a smaller current density of $j_{m}=12.5$ A g${}^{-1}$. It is noted that $C_{m}$ of NPG/PPy(30 nm) abruptly dropped at $j_{m}=7.5$ A g${}^{-1}$ even the specimen was at a pristine state. The cycling performances of NPG/PPy hybrid materials were examined under strain values of 0% and 15%. Current densities in the orange rectangular area (Figure 3c) were elaborately chosen to attain comparable $C_{m}$ for each specimen. The results are compiled in Figure 3d. It is found that $C_{m}$ obtained under a deformation of 15% are smaller than those of 0% for both NPG/PPy(10 nm) and NPG/PPy(30 nm) at all explored current densities over the studied cycles. Moreover, for a given specimen, comparable $C_{m}$ values were obtained at different $j_{m}$ values under the same deformation except in the cases of 15%-deformed NPG/PPy(30 nm). $C_{m}$ values at the beginning and end of the cycling course are summarized in Table 1. One can find that in comparison with the non-deformed specimens, the durability of 15%-deformed ones was substantially impaired.

### 3.4. Electrochemical Impedance Spectroscopy

**Figure 4:**
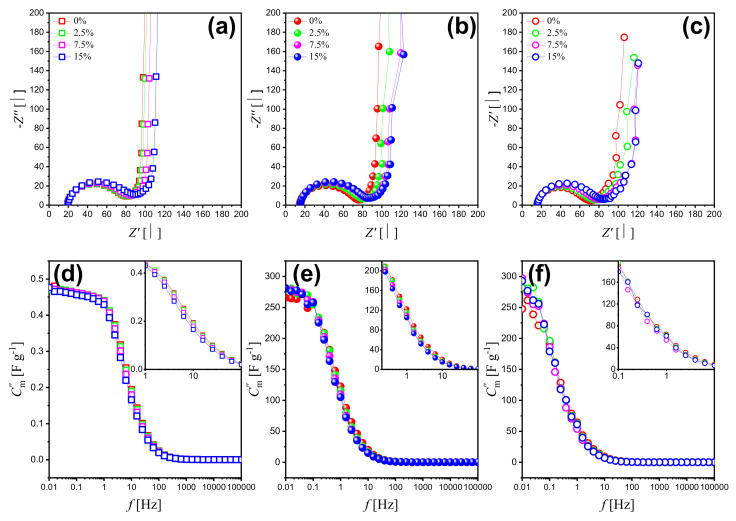
(**a**–**c**) Nyquist plots of bare NPG, NPG/PPy(10 nm), and NPG/PPy(30 nm) under various deformations and their corresponding Bode Plots (**d**–**f**) with capacitance calculated from the imaginary part of impedance. Open square, closed symbol, and open circle represent NPG, NPG/PPy(10 nm), and NPG/PPy(30 nm), respectively. The color conventions indicate the deformation. Insets in (**d**–**f**) show Bode Plots of middle frequency range at a higher magnification.

The electrochemical properties of NPG and NPG/PPy hybrid materials were also explored by electrochemical impedance spectroscopy (EIS).Figure 4a–c plot the Nyquist diagrams of bare NPG and NPG/PPy hybrid materials under various deformations. It has been shown that all of the curves consist of a distinct semicircle at high frequencies and a straight line at lower frequencies. The negligibly small discrepancies in the interceptions between Nyquist curves and real part-axes demonstrate that the bulk electrolyte resistance and any contact resistance keep unchanged under deformation for all the investigated specimens. This is also evidenced by the fitting parameter $R_{0}$ shown in Table 2. Yet, the radius was observed to increase with deformation. Despite the disturbances, the lower frequency region shows (quasi-) straight lines, which shift positively with deformation.

Capacitance contributes to both the real and imaginary parts of the impedance. However, the influence of the capacitance on the real part is negligible in a broad frequency range while it is decisive of the imaginary part. Accordingly, the imaginary part of the impedance is in focus as shown in Figure 4d–f. The frequency-dependent capacitance was calculated from the imaginary part of the impedance as [39]:

(1) $$C^{\prime \prime }{}_{m}\left(f\right)=-\frac{1}{2\pi fZ^{\prime \prime }m}$$

where *f*, $Z^{\prime \prime }$, and *m* represent frequency, imaginary part of the impedance, and mass of active material addressed in the following part. As can be seen in Figure 4, $C_{m}^{\prime \prime }-$ *f* curves of bare NPG and NPG/PPy hybrid materials showed common features and are divided into three parts: (1) The part at high frequency range (larger than 100 Hz), where the capacitance seems to be frequency independent and contributes negligibly at various compression strain values. (2) The part at the frequency range of 100 Hz∼0.1 Hz, where the capacitance increases linearly along with the decreasing frequency. In this part, the capacitance value of NPG/PPy hybrids improved compared with that of bare NPG, which is consistent with CV and GCD results. Moreover, the capacitance values slightly decrease at increasing deformation and a given frequency value. (3) The part at the low frequency range (lower than 0.1 Hz), where the capacitance nearly approaches the saturation. Despite some disturbances in NPG/PPy hybrid materials, the dependence of capacitance on the deformation is similar to that observed for the middle frequency range.

## 4. Discussion

The present work studies the in situ electrochemical behavior of NPG/PPy hybrid materials subjected to compressive deformations in a range from 0% to 15%. The discussion starts with the current responses of non-deformed NPG and NPG/PPy. Despite the similar rectangular profiles, NPG and NPG/PPy store charges in different ways. In the explored potential window, anions (ClO${}_{4}$${}^{-}$ in this work) are electrostatically adsorbed onto the gold ligament surface, following the capacitive charging mechanism [2,40,41]. On the other hand, anions migrate into the PPy matrix to maintain the charge neutrality when PPy is oxidized, which is the characteristic of the pseudocapacitive charging mechanism [41,42,43]. The electrochemical responses were normalized to the weight of the active material, *m*. Since the surface atoms take up a high percentage of the overall atoms in nano-scaled objects [44], *m* is equal to $m_{\mathrm{NPG}}$ for the bare NPG. Yet, when NPG is coated by a second active material, say, MnO${}_{2}$ and PPy, the current response mainly originates from the reactions between active material matrix and bulk solution, whereas the electrosorption at NPG/MnO${}_{2}$ and the NPG/PPy interface is negligibly small [45,46]. To this end, *m* = $m_{\mathrm{PPy}}$ for NPG/PPy hybrid materials. In this work, $m_{\mathrm{NPG}}$ is one order of magnitude higher than $m_{\mathrm{PPy}}$. Moreover, it has been shown that more charge carriers participate in the pseudocapacitive charging process compared to those in the double layer process [47,48]. Therefore, NPG/PPy hybrid materials yield a much higher specific current density, $j_{m}$ (Figure 2a), and specific capacitance, $C_{m}$ (Figure 2d), compared with bare NPG. The resulting current responses (Figure S7) and $m_{\mathrm{PPy}}$ synergetically determine higher $C_{m}$ and $j_{m}$ for NPG/PPy(10 nm) compared to those of NPG/PPy(30 nm). The same phenomenon has also been revealed in the literature [17,27,29], where the lower $C_{m}$ values of materials with thicker deposited layers are ascribed to the smaller surface area exposed to the electrolyte and only partial PPy take part in the reactions. A similar phenomenon was also observed in the GCD results, where NPG/PPy(10 nm) processed a higher $C_{m}$ in comparison with NPG/PPy(30 nm) (Figure 3c).

The availability of active material is also influenced by the kinetics of electrolyte transport. The current density increases with scan rates (Figure 2c), indicating excellent electronic conductivity of the NPG/PPy [49]. However, the deviation of the CV curve from a rectangular shape (Figure 2c) and the drop of $C_{m}$ at the faster scan rates (Figure 2d) observed for both NPG/PPy specimens suggest the dominant role of mass transport in the NPG/PPy/electrolyte system [50,51]. On the other hand, the linear I−v relationship (Figure S2b) and the fairly unvaried $C_{m}$ over studied scan rates (inset in Figure 2d) manifest the adsorption-controlled process in the NPG/electrolyte system. In the case of GCD, when the discharging current density increases, the incremental IR drops (Figure 3b and Figure S6) and the degressive $C_{m}$ (Figure 3c and Figure S5c) demonstrate insufficient active material involved in the reaction, especially, for NPG/PPy.

Till now, it has been clarified that the electrochemical performance of NPG and NPG/PPy under zero deformation, where the microstructure is kept unchanged (neglecting the electrochemical process-induced expansion and contraction with amplitudes of ∼10${}^{-4}$ [18,52]), is substantially determined by the amount of involved active material, which is closely associated with the kinetics of the electrolyte in the confined microstructure. Below, the impact of the compressive deformation-induced microstructure change on the mass transport and the accompanying electrochemical behavior are discussed.

For bare NPG, comparable CV curves (inset in Figure 2a) and GCD curves (Figure S5a) were obtained under deformations of 0% and 15%. This demonstrates that the capacitive charging and adsorption processes in NPG/electrolyte are negligibly impacted in the deformed microstructure. In the case of NPG/PPy hybrid materials, however, the CV curves under deformation of 15% deviate from those of 0%. Moreover, despite the CV curves obtained under 2.5%∼15% being similar, the corresponding $C_{m}$ values were observed to decrease with deformation (Figure 2d), indicating that the pseudocapacitive charging and mass transport are substantially influenced in the compacted specimens. Besides, the descending $C_{m}$ values evaluated from GCD results under the ascending deformations (Figure 3c) also verify the aforementioned findings.

As is widely acknowledged, semi-circles at a high frequency regime in the Nyquist plots reflect the charge transfer resistance between the electrode and solution. The semi-circle in the bare NPG can be ascribed to the oxidation of residual Ag at the applied potential of 0.5 V [53,54]. For the NPG/PPy hybrid materials, it can be ascribed to the ion transfer at the PPy/solution boundary. The larger $R_{\mathrm{ct}}$ obtained from EIS data indicate larger charge transfer resistance and slower mass transfer kinetics. It is also reported that the decreased availability of active material for electrolyte impregnation will induce larger impedance [55], implying the significant role of deformation-caused microstructure change on the properties of electrode material. The behavior of the interface in the oxidized state in both specimens was similar. Towards the high and medium frequencies, the role of diffusion-like and charge transfer steps increases with the deformation, which also explain the current response reduction under different deformation in the CV tests.

The overall current output comprises surface-controlled (capacitive) and diffusion-controlled components. According to the peak current (*i*) in CV curves at various scan rates (*v*), they can be identified via the following equation [56,57]:

(2) $$i=av^{b}$$

where *a* and *b* are adjustable parameters. The slope of log(*i*) vs. log(*v*) plot gives *b*. Pure capacitive dominated processes and diffusion dominated processes are characterized by a *b* of 1 and 0.5, respectively. When *b* lies between 0.5 and 1, a mixed capacitive-diffusion controlled process is identified. At a given potential (*V*), the two contributions can be distinguished according to the following equation [56,57]:

(3) $$i\left(V\right)=k_{1}v+k_{2}v^{1/2}$$

where $k_{1}v$ and $k_{2}v^{1/2}$ respectively represent surface capacitive current and diffusion-limited current. This equation can also be rearranged to:

(4) $$i\left(V\right)/v^{1/2}=k_{1}v^{1/2}+k_{2}$$

to this end, $k_{1}$ and $k_{2}$ can be evaluated from the slope and intercept from $i\left(V\right)/v^{1/2}$ vs. the $v^{1/2}$ plot, respectively.

**Figure 5:**
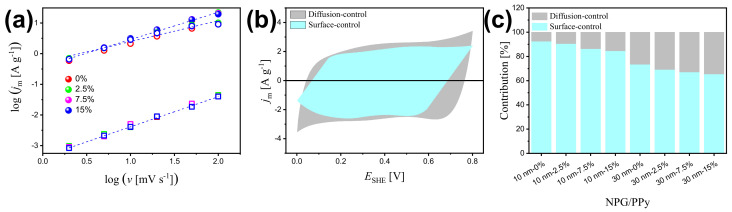
(**a**) Linear fitting of the logarithm of peak current density, log($j_{m}$), as a function of logarithm of scan rates, log(*v*), for bare NPG, NPG/PPy(10 nm), and NPG/PPy(30 nm) under various deformations. Open square, closed symbol, and open circle represent NPG, NPG/PPy(10 nm), and NPG/PPy(30 nm), respectively. The color conventions indicate the deformation. (**b**) Voltammetric response of non-deformed NPG/PPy(10 nm)at a scan rate of 10 mV s${}^{-1}$. The contributions from surface-controlled and diffusion-controlled processes are determined from Equation (4). (**c**) Capacitance contribution at *v* = 10 mV s${}^{-1}$ for NPG/PPy specimens under different deformations.

Figure 5a presents the linear regressions of log($j_{m}$) vs. log(*v*), yielding *b* = 0.98 ($R^{2}$ = 0.998), *b* = 0.90 ($R^{2}$ = 0.998), and *b* = 0.70 ($R^{2}$ = 0.996) for non-deformed NPG, NPG/PPy(10 nm), and NPG/PPy(30 nm), respectively. This suggests a nearly pure capacitive process in bare NPG and mixed capacitive-diffusion dominated processes in NPG/PPy specimens (Figure 5b). Under deformation of 15%, a comparable *b* value (0.98, $R^{2}$ = 0.999) was obtained for NPG, implying that the surface capacitive process is free from deformation and the influence of surface area loss caused by the gold ligaments contact (see discussion below) on the capacitance of bare NPG can be neglected. On the other hand, smaller slopes of *b* = 0.88 ($R^{2}$ = 0.993) and *b* = 0.67 ($R^{2}$ = 0.990) are estimated respectively for 15%-deformed NPG/PPy(10 nm) and NPG/PPy(30 nm), leading to an improved contribution of diffusion process under deformation (Figure 5c).

The increased diffusion-controlled effect is related to the intrinsic microstructure change of the material under compression. Higher electrochemical performance has been reported in nanoporous materials with hierarchical microstructure due to the improved diffusion of electrolyte in large pores [58,59,60,61]. In the context of nanoporous metal with monolithic-scale pore distribution, the mass transport should be different from that in hierarchical nanoporous materials. An important figure of merit to characterize the effect of microstructure of a porous electrode on its effective transport is tortuosity, τ [62,63]. In terms of geometrics, τ is the fraction of the shortest pathway for the electrolyte to flow through the porous structure and the Euclidean distance between the start and end points of this pathway. According to the Bruggeman equation, the tortuosity-microstructure relationship is described as [63,64]:

(5) $$\tau^{2}=\epsilon^{-0.5}$$

where ϵ is the porosity. Taking the porosity of NPG of ϵ∼ 0.7 and PPy film density of $\rho_{\mathrm{PPy}}=1.5$ g/cm${}^{3}$ [65,66], τ was estimated to be 1.08, 1.10, and 1.17 for NPG, NPG/PPy(10 nm), and NPG/PPy(30 nm), respectively.

As has been reported, the change in Poisson’s ratio of nanoporous gold with ligament size of 120∼180 nm, which embraces the ligament size in the present work, was smaller than 0.025 when the NPG was compressed up to 20% [67]. In other words, such a small transverse deformation mainly led to the densification of NPG in the loading axis under compression, giving rise to a smaller ϵ and, consequently, a larger τ. Under a deformation of 15%, τ of NPG, NPG/PPy(10 nm), and NPG/PPy(30 nm) increased to 1.13, 1.15, and 1.22, respectively. Tortuosity has also been reported to be related to the diffusion coefficient [63,68,69]:

(6) $$\tau^{2}=\frac{\epsilon }{D{}_{\mathrm{eff}}}D_{\mathrm{bulk}}$$

where $D_{\mathrm{bulk}}$ and $D_{\mathrm{eff}}$ represent bulk diffusion coefficient and effective diffusion coefficient, respectively. To this end, $D_{\mathrm{eff}}$ has been revealed to decrease by ∼21% in all 15%-deformed NPG and NPG/PPy hybrid materials. The limited mass transport might be attributed to the microstructure change under deformation. Simulations of compression of random networks demonstrates that the struts along the load tend to be bended, while the ones perpendicular to the load will be stretched [70]. Given this, the adjacent ligaments in the deformed network are anticipated to encounter each other, as indicated by the red ovals in the SEM images (Figure 1d–f), giving rise to a larger amount of connections. With increasing compressing of the specimen, its connectivity density will increase and result in a more complex network. Finally, this leads to a restrained mass transport.

## 5. Conclusions

This work explores the electrochemical behavior of the NPG/PPy supercapacitor under various compression deformation states. The macroscopic specimen, synthesized by dealloying and electropolymerization, consists of billions of nano-scaled ligaments and pores. The unique bi-continuous network provides numerous pathways for electrolyte and active sites, meanwhile exhibiting considerable ability against the external load, predestining NPG/PPy as a promising model material to study the electrochemical performance under compressive deformation. Electrochemical measurements of bare NPG, NPG/PPy(10 nm), and NPG/PPy(30 nm) were carried out in situ under different compression strains in the range of 0 to 15%, respectively. NPG/PPy(10 nm) possesses larger specific capacitance, $C_{m}$, as compared with NPG/PPy(30 nm) and bare NPG. The $C_{m}$ is found to decrease with deformation at studied scan rates and discharging current densities for all explored specimens. NPG/PPy hybrid materials show high stability with 70% capacitance retained after 4000 cycles. Moreover, the charge transfer resistance, $R_{\mathrm{ct}}$, increases with deformation. This electrochemical behavior of NPG/PPy is due to the locally reduced mass transport of electrolyte caused by the formation of new connections between the neighboring ligaments under the application of compression loads. The fundamental understanding of the effect of deformation on the performance of energy storage materials revealed in this study paves the way for their practical application in wearable devices.

## Figures and Tables

**Table 1:** Specific capacitance, $C_{m}$, of NPG/PPy hybrid materials at the beginning and end of GCD cycling under various deformations and current densities. The data in the table are in agreement with the data points shown in Figure 3d.

Specimen	Deformation (%)	Current Density (A g${}^{-1}$)	$C_{m0}$ (F g${}^{-1}$) ^a^	$C_{m1}$ (F g${}^{-1}$) ^b^	Retention Ratio (%)
NPG/PPy(10 nm)	0	7.5	260	195	75
	15	5	170	115	67.6
	15	7.5	170	125	73.5
NPG/PPy(30 nm)	0	2.5	270	215	79.6
	0	5	280	280	85.7
	15	2.5	210	150	71.4
	15	5	260	200	76.9

^a^ Specific capacitance obtained at the beginning of GCD cycling. ^b^ Specific capacitance obtained at the end of GCD cycling.

**Table 2:** Fitting parameters of bare NPG, NPG/PPy(10 nm), and NPG/PPy(30 nm) under various deformations based on the equivalent circuit in Figure S8.

	NPG		NPG/PPy(10 nm)		NPG/PPy(30 nm)	
**Deformation (%)**	$R_{0}$ **(** Ω **)**	**$R_{\mathrm{ct}}$ (Ω)**	$R_{0}$ **(** Ω **)**	$R_{\mathrm{ct}}$ **(** Ω **)**	$R_{0}$ **(** Ω **)**	$R_{\mathrm{ct}}$ **(** Ω **)**
0	17.75	65.19	13.57	62.86	14.42	60.82
2.5	17.91	66.37	14.16	67.23	14.42	60.82
7.5	18.05	69.51	14.32	71.83	14.73	64.59
15	18.15	74.56	14.51	72.54	14.96	68.43

## Data Availability

The data presented in this study are available on request from the corresponding author.

## Supplementary Materials

The following supporting information can be downloaded at: https://www.mdpi.com/article/10.3390/nano12132149/s1, Capacitance calculation method; Figure S1: EDS spectrum of NPG; Figure S2: CV curves of NPG at various scan rates [36]; Figure S3: Loading-unloading curves of NPG and NPG/PPy specimens; Figure S4: CV curves of NPG/PPy(10 nm) at different potential ranges; Figure S5: GCD curves of NPG; Figure S6: GCD curves of NPG/PPy(30 nm) at different current densities; Figure S7: CV curves of NPG/PPy(10 nm) and NPG/PPy(30 nm) at 5 mV s${}^{-1}$; Figure S8: Equivalent circuit for EIS fitting.

## References

[1] Sun H., Zhu J., Baumann D., Peng L., Xu Y., Shakir I., Huang Y., Duan X. (2019). Hierarchical 3D electrodes for electrochemical energy storage. Nat. Rev. Mater..

[2] Lang X.Y., Yuan H.T., Iwasa Y., Chen M.W. (2011). Three-dimensional nanoporous gold for electrochemical supercapacitors. Scr. Mater..

[3] Wen S., Jung M., Joo O.S., Mho S.I. (2006). EDLC characteristics with high specific capacitance of the CNT electrodes grown on nanoporous alumina templates. Curr. Appl. Phys..

[4] Choi C., Ashby D.S., Butts D.M., DeBlock R.H., Wei Q., Lau J., Dunn B. (2020). Achieving high energy density and high power density with pseudocapacitive materials. Nat. Rev. Mater..

[5] Liu T., Finn L., Yu M., Wang H., Zhai T., Lu X., Tong Y., Li Y. (2014). Polyaniline and Polypyrrole Pseudocapacitor Electrodes with Excellent Cycling Stability. Nano Lett..

[6] Hu C.C., Chang K.H., Lin M.C., Wu Y.T. (2006). Design and tailoring of the nanotubular arrayed architecture of hydrous RuO2 for next generation supercapacitors. Nano Lett..

[7] Ragupathy P., Vasan H., Munichandraiah N. (2007). Synthesis and characterization of nano-MnO2 for electrochemical supercapacitor studies. J. Electrochem. Soc..

[8] Xiong X., Ding D., Chen D., Waller G., Bu Y., Wang Z., Liu M. (2015). Three-dimensional ultrathin Ni(OH)2 nanosheets grown on nickel foam for high-performance supercapacitors. Nano Energy.

[9] Detsi E., Onck P., De Hosson J.T.M. (2013). Metallic Muscles at Work: High Rate Actuation in Nanoporous Gold/Polyaniline Composites. ACS Nano.

[10] Zhang J., Kong L.B., Li H., Luo Y.C., Kang L. (2010). Synthesis of polypyrrole film by pulse galvanostatic method and its application as supercapacitor electrode materials. J. Mater. Sci..

[11] Vernitskaya T.V., Efimov O.N. (1997). Polypyrrole: A conducting polymer; its synthesis, properties and applications. Russ. Chem. Rev..

[12] Chen Y., Du L., Yang P., Sun P., Yu X., Mai W. (2015). Significantly enhanced robustness and electrochemical performance of flexible carbon nanotube-based supercapacitors by electrodepositing polypyrrole. J. Power Sources.

[13] Hu Y., Zhao Y., Li Y., Li H., Shao H., Qu L. (2012). Defective super-long carbon nanotubes and polypyrrole composite for high-performance supercapacitor electrodes. Electrochim. Acta.

[14] Parayangattil Jyothibasu J., Chen M.Z., Lee R.H. (2020). Polypyrrole/carbon nanotube freestanding electrode with excellent electrochemical properties for high-performance all-solid-state supercapacitors. ACS Omega.

[15] Yang X., Lin Z., Zheng J., Huang Y., Chen B., Mai Y., Feng X. (2016). Facile template-free synthesis of vertically aligned polypyrrole nanosheets on nickel foams for flexible all-solid-state asymmetric supercapacitors. Nanoscale.

[16] Wang Z., Ning S., Liu P., Ding Y., Hirata A., Fujita T., Chen M. (2017). Tuning Surface Structure of 3D Nanoporous Gold by Surfactant-Free Electrochemical Potential Cycling. Adv. Mater..

[17] Liu P., Ge X., Wang R., Ma H., Ding Y. (2009). Facile Fabrication of Ultrathin Pt Overlayers onto Nanoporous Metal Membranes via Repeated Cu UPD and in Situ Redox Replacement Reaction. Langmuir.

[18] Li J., Markmann J., Weissmüller J., Mameka N. (2021). Nanoporous gold-polypyrrole hybrid electrochemical actuators with tunable elasticity. Acta Mater..

[19] Wang K., Weissmüller J. (2013). Composites of Nanoporous Gold and Polymer. Adv. Mater..

[20] Hodge A.M., Hayes J.R., Caro J.A., Biener J., Hamza A.V. (2006). Characterization and Mechanical Behavior of Nanoporous Gold. Adv. Eng. Mater..

[21] Biener J., Hodge A.M., Hamza A.V., Hsiung L.M., Satcher J.H. (2005). Nanoporous Au: A high yield strength material. J. Appl. Phys..

[22] Jin H.J., Weissmüller J., Farkas D. (2018). Mechanical response of nanoporous metals: A story of size, surface stress, and severed struts. MRS Bull..

[23] Ding Y., Erlebacher J. (2003). Nanoporous metals with controlled multimodal pore size distribution. J. Am. Chem. Soc..

[24] Wang K., Stenner C., Weissmüller J. (2017). A nanoporous gold-polypyrrole hybrid nanomaterial for actuation. Sens. Actuators B Chem.

[25] Gao P., Qian P., Qiao L., Volinsky A.A., Su Y. (2019). Atomic force microscopy study of nanoporous gold surface and electrochemical properties. Scr. Mater..

[26] Roschning B., Weissmüller J. (2020). Nanoporous-Gold-Polypyrrole Hybrid Materials for Millimeter-Sized Free Standing Actuators. Adv. Mater. Interfaces.

[27] Meng F., Ding Y. (2011). Sub-Micrometer-Thick All-Solid-State Supercapacitors with High Power and Energy Densities. Adv. Mater..

[28] Hou Y., Chen L., Liu P., Kang J., Fujita T., Chen M. (2014). Nanoporous metal based flexible asymmetric pseudocapacitors. J. Mater. Chem. A.

[29] Hou Y., Chen L., Zhang L., Kang J., Fujita T., Jiang J., Chen M. (2013). Ultrahigh capacitance of nanoporous metal enhanced conductive polymer pseudocapacitors. J. Power Sources.

[30] Hou Y., Chen L., Hirata A., Fujita T., Chen M. (2016). Non-aqueous nanoporous gold based supercapacitors with high specific energy. Scr. Mater..

[31] Hou Y., Zhang L., Chen L.Y., Liu P., Hirata A., Chen M.W. (2014). Raman characterization of pseudocapacitive behavior of polypyrrole on nanoporous gold. Phys. Chem. Chem. Phys..

[32] Murali S., Quarles N., Zhang L.L., Potts J.R., Tan Z., Lu Y., Zhu Y., Ruoff R.S. (2013). Volumetric capacitance of compressed activated microwave-expanded graphite oxide (a-MEGO) electrodes. Nano Energy.

[33] Li X., Rong J., Wei B. (2010). Electrochemical Behavior of Single-Walled Carbon Nanotube Supercapacitors under Compressive Stress. ACS Nano.

[34] Zhong Y., Markmann J., Jin H.J., Ivanisenko Y., Kurmanaeva L., Weissmüller J. (2014). Crack Mitigation during Dealloying of Au25Cu75. Adv. Eng. Mater..

[35] Trasatti S., Petrii O. (1991). Real surface area measurements in electrochemistry. Pure Appl. Chem..

[36] Zhumaev U.E., Lai A.S., Pobelov I.V., Kuzume A., Rudnev A.V., Wandlowski T. (2014). Quantifying perchlorate adsorption on Au (1 1 1) electrodes. Electrochim. Acta.

[37] Lewis T.W., Wallace G.G., Kim C.Y., Kim D.Y. (1997). Studies of the overoxidation of polypyrrole. Synth. Met..

[38] Li J. (2021). Electrochemically Tunable Mechanical Behavior of Bulk Nanoporous Gold/Polypyrrole. Ph.D. Thesis.

[39] Taberna P.L., Simon P., Fauvarque J.F. (2003). Electrochemical Characteristics and Impedance Spectroscopy Studies of Carbon-Carbon Supercapacitors. J. Electrochem. Soc..

[40] Silva Olaya A.R., Zandersons B., Wittstock G. (2020). Restructuring of nanoporous gold surfaces during electrochemical cycling in acidic and alkaline media. ChemElectroChem.

[41] Gogotsi Y., Penner R.M. (2018). Energy Storage in Nanomaterials - Capacitive, Pseudocapacitive, or Battery-like?. ACS Nano.

[42] Costentin C., Porter T.R., Savéant J.M. (2017). How do pseudocapacitors store energy? Theoretical analysis and experimental illustration. ACS Appl. Mater. Interfaces.

[43] Brinker M., Dittrich G., Richert C., Lakner P., Krekeler T., Keller T.F., Huber N., Huber P. (2020). Giant electrochemical actuation in a nanoporous silicon-polypyrrole hybrid material. Sci. Adv..

[44] Birringer R., Gleiter H., Klein H.P., Marquardt P. (1984). Nanocrystalline materials an approach to a novel solid structure with gas-like disorder?. Phys. Lett. A.

[45] Lang X., Hirata A., Fujita T., Chen M. (2011). Nanoporous metal/oxide hybrid electrodes for electrochemical supercapacitors. Nat. Nanotechnol..

[46] Roschning B., Weissmüller J. (2019). Stress-charge coupling coefficient for thin-film polypyrrole actuators – Investigation of capacitive ion exchange in the oxidized state. Electrochim. Acta.

[47] Escobar-Teran F., Arnau A., Garcia J.V., Jiménez Y., Perrot H., Sel O. (2016). Gravimetric and dynamic deconvolution of global EQCM response of carbon nanotube based electrodes by Ac-electrogravimetry. Electrochem. Commun..

[48] Wu C., Wang J., Bai Y., Li X. (2020). Significant effect of cations on polypyrrole cycle stability. Solid State Ion..

[49] Dong C., Wang Y., Xu J., Cheng G., Yang W., Kou T., Zhang Z., Ding Y. (2014). 3D binder-free Cu2O@Cu nanoneedle arrays for high-performance asymmetric supercapacitors. J. Mater. Chem. A.

[50] Batchelor-McAuley C., Gonçalves L.M., Xiong L., Barros A.A., Compton R.G. (2010). Controlling voltammetric responses by electrode modification; using adsorbed acetone to switch graphite surfaces between adsorptive and diffusive modes. Chem. Commun..

[51] Kumar A., Gonçalves J.M., Selva J.S.G., Araki K., Bertotti M. (2019). Correlating Selective Electrocatalysis of Dopamine and Ascorbic Acid Electrooxidation at Nanoporous Gold Surfaces with Structural-Defects. J. Electrochem. Soc..

[52] Liu L.Z., Mameka N., Markmann J., Jin H.J., Weissmüller J. (2019). Surface-driven actuation: Sign reversal under load and surface load-memory effect. Phys. Rev. Mater..

[53] Krekeler T., Straßer A.V., Graf M., Wang K., Hartig C., Ritter M., Weissmüller J. (2017). Silver-rich clusters in nanoporous gold. Mater. Res. Bull..

[54] Rouya E., Cattarin S., Reed M.L., Kelly R.G., Zangari G. (2012). Electrochemical Characterization of the Surface Area of Nanoporous Gold Films. J. Electrochem. Soc..

[55] Shodiev A., Primo E.N., Chouchane M., Lombardo T., Ngandjong A.C., Rucci A., Franco A.A. (2020). 4D-resolved physical model for Electrochemical Impedance Spectroscopy of Li(Ni1-x-yMnxCoy)O2-based cathodes in symmetric cells: Consequences in tortuosity calculations. J. Power Sources.

[56] Forghani M., Donne S.W. (2018). Method comparison for deconvoluting capacitive and pseudo-capacitive contributions to electrochemical capacitor electrode behavior. J. Electrochem. Soc..

[57] Da Rocha M., Dunn B., Rougier A. (2019). Faradaic and/or capacitive: Which contribution for electrochromism in NiO thin films cycled in various electrolytes?. Sol. Energy Mater. Sol. Cells.

[58] Cheng C., Grant P.S., Lührs L. (2020). Electrochemical Mechanics of Metal Thin Films: Charge-Induced Reversible Surface Stress for Actuation. Adv. Electron. Mater..

[59] Cheng C., Lührs L., Krekeler T. (2021). Simultaneous Enhancement of Actuation Strain and Mechanical Strength of Nanoporous Ni-Mn Actuators. Adv. Electron. Mater..

[60] Qi Z., Weissmüller J. (2013). Hierarchical nested-network nanostructure by dealloying. ACS Nano.

[61] Gaya C., Yin Y., Torayev A., Mammeri Y., Franco A.A. (2018). Investigation of bi-porous electrodes for lithium oxygen batteries. Electrochim. Acta.

[62] Chung D.W., Ebner M., Ely D.R., Wood V., Edwin García R. (2013). Validity of the Bruggeman relation for porous electrodes. Model. Simul. Mater. Sci. Eng..

[63] Tjaden B., Brett D.J.L., Shearing P.R. (2018). Tortuosity in electrochemical devices: A review of calculation approaches. Int. Mater. Rev..

[64] Bruggeman D.A.G. (1935). Berechnung verschiedener physikalischer Konstanten von heterogenen Substanzen. I. Dielektrizitätskonstanten und Leitfähigkeiten der Mischkörper aus isotropen Substanzen. Ann. Phys..

[65] Hepel M. (1998). The electrocatalytic oxidation of methanol at finely dispersed platinum nanoparticles in polypyrrole films. J. Electrochem. Soc..

[66] Hallik A., Roosalu K., Mändar H., Joosu L., Marandi M., Tamm J. (2015). Thickness dependence of the porosity of PPy/DDS films. Eur. Polym. J..

[67] Lührs L., Soyarslan C., Markmann J., Bargmann S., Weissmüller J. (2016). Elastic and plastic Poisson’s ratios of nanoporous gold. Scr. Mater..

[68] Ebner M., Wood V. (2014). Tool for Tortuosity Estimation in Lithium Ion Battery Porous Electrodes. J. Electrochem. Soc..

[69] Cooper S.J., Bertei A., Shearing P.R., Kilner J.A., Brandon N.P. (2016). TauFactor: An open-source application for calculating tortuosity factors from tomographic data. SoftwareX.

[70] Burg M.W.D.V.D., Shulmeister V., Geissen E.V.D., Marissen R. (1997). On the Linear Elastic Properties of Regular and Random Open-Cell Foam Models. J. Cell. Plast..

