# Supplementary material for: Electrochemical Behavior of Nanoporous Gold/Polypyrrole Supercapacitor under Deformation
Source: Nanomaterials (Basel). 2022 Jun 22;12(13):2149. doi: 10.3390/nano12132149 (PMC9267961; doi:10.3390/nano12132149)
Supplement: Supplementary file 1 [file nanomaterials-12-02149-s001.zip › nanomaterials-1775724-supplementary.pdf]

nanomaterials

Supplementary Materials

Electrochemical Behavior of Nanoporous Gold/Polypyrrole Supercapacitor under Deformation

Jie Li [1,2,*,‡], Liangyu Li [2,‡], Peng Jia [3] and Ilya V. Okulov [4,5,*]

[1] Materials Mechanics, Institute of Materials Research, Helmholtz-Zentrum Hereon, Max-Planck-Str.1, 21502 Geesthacht, Germany
[2] Department of Mechanical and Aerospace Engineering, Hong Kong University of Science and Technology, Clear Water Bay, Hong Kong, China; liliangyu@ust.hk
[3] State Key Laboratory of Biobased Material and Green Papermaking, Key Laboratory of Pulp and Paper Science & Technology of Ministry of Education/Shandong Province, Faculty of Light Industry, Qilu University of Technology (Shandong Academy of Sciences), 250353 Jinan, China; skl_jiapeng@163.com
[4] Leibniz Institute for Materials Engineering-IWT, Badgasteiner Str. 3, 28359 Bremen, Germany
[5] Faculty of Production Engineering, University of Bremen, Badgasteiner Str. 1, 28359 Bremen, Germany
‡ These authors contributed equally to this work.
* Correspondence: jieli@ust.hk or jie.li_x@hotmail.com (J.L.); i.okulov@iwt.uni-bremen.de (I.V.O)

Calculations

The specific capacitance can be calculated from a cyclic voltammogram (CV) according to the following equation [48]:

$$C_m = \frac{\int_{V_0}^{V_1} I(V)dV}{mv(V_1 - V_0)} \quad (1)$$

where C_m is the specific capacitance (F g^{-1}), m is the weight (g) of the electroactive material, v is the scan rate (mV s^{-1}), V_1 is highest potential (V), V_0 is the lowest potential, and $I(V)$ is the current response (A) with respect to the potential (V).

Alternatively, the C_m can also be evaluated based on the galvanostatic charge-discharge (GCD) curves via the following equation [12]:

$$C_m = \frac{i \cdot \Delta t}{m \cdot \Delta V} \quad (2)$$

where i is the constant discharge current (A), Δt is the discharge time, ΔV is the discharge potential range (V) excluding the IR drop, and m is the mass of the electroactive material (g).

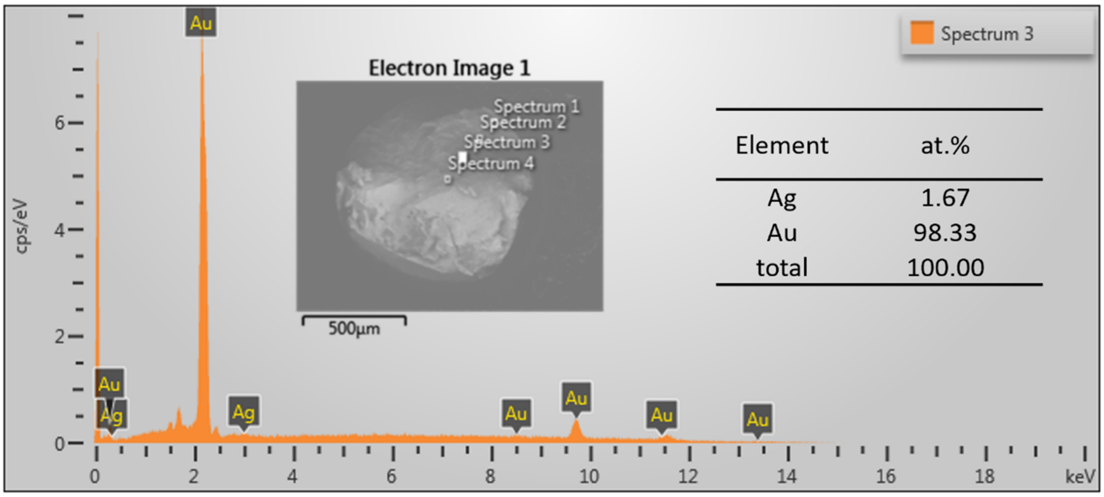

Figure S1. A representative energy dispersive spectroscopy (EDS) spectrum showing the composition of the central part of the as-dealloyed NPG shown in the inset figure. The inset table lists the content of the detected elements.

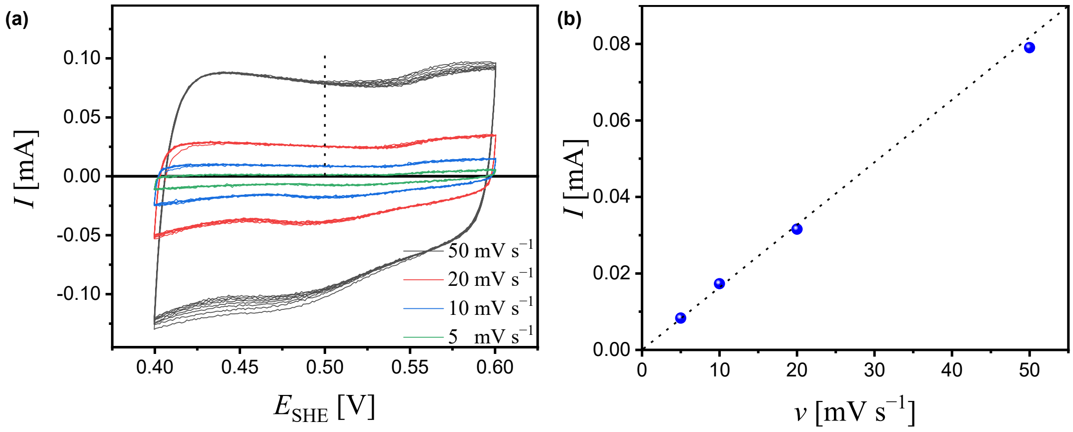

Figure S2. (**a**) Cyclic voltammogram (CV) of bare NPG at various scan rates of 5 ~ 50 mV s^{-1}. Eight successive cycles are shown in the figure; the traces at a scan rate < 50mV s^{-1} are superimposed. (**b**) Current, *I*, of middle of the applied potential range (indicated by the dotted line in (**a**)) versus scan rate, *v*. The slope of the linear regression (dotted line in (**b**)) gives rise to the capacitance.

The current response within the narrow potential regime shows a quasi-rectangular profile (Figure S2), indicating the capacitive charging-discharging mechanism. The surface area of NPG can be evaluated based on the capacitance ratio method [35], where the slope of linear regression gives rise to the capacitance of the specimen. A specific capacitance of 20 uF cm^{-2} for gold plane in 0.1 M $HClO_4$ has been reported in reference [70], and the volumetric surface area is then determined to be ~80 cm^2 mm^{-3} which is used to predict the ligament size as described in the main text.

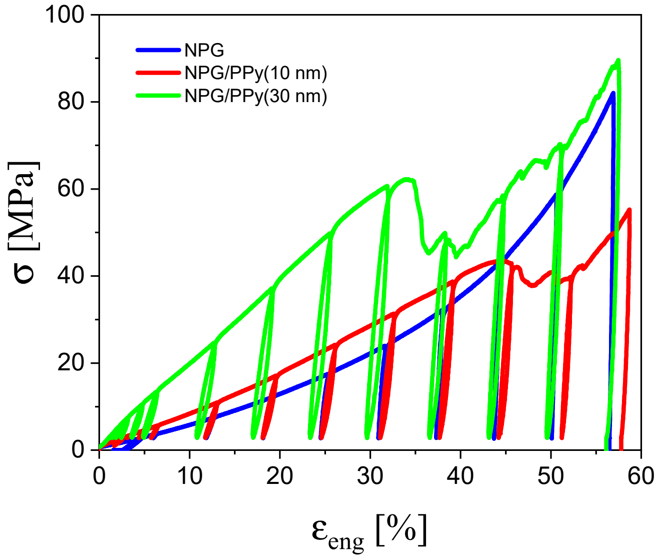

Figure S3. Loading-unloading curves of NPG, NPG/PPy (10 nm), and NPG/PPy (30 nm).

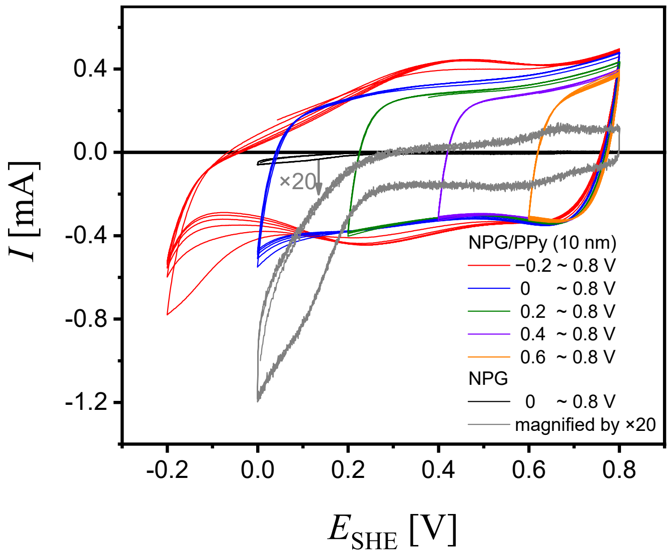

Figure S4. CV curves of NPG/PPy (10 nm) and NPG in various potential regimes. CV curve of NPG (black) is magnified by 20 times (gray line). All CV curves are recorded at a scan rate of 5 mV s^{-1} in 0.1 M $HClO_4$. Six cycles are shown, traces obtained within 0 ~ 0.8 V superimposed for both NPG and NPG/PPy (10 nm).

Figure S4 illustrates the CV curves of NPG/PPy (10 nm) and bare NPG in various potential windows. Clearly, a quasi-rectangular profile is seen at 0 ~ 0.8 V for NPG/PPy (10 nm), suggesting the same pseudo-capacitive charging-discharging processes within the explored potential windows for NPG/PPy. However, distorted CV curves are obtained when the lower potential limit is extended to −0.2 V. Therefore, the electrochemical properties of NPG/PPy hybrids are studied in 0 ~ 0.8 V in this work. Yet, the negative current values below 0.2 V might invoke the hydrogen evolution on the NPG ligament; therefore, NPG was studied within a potential range of 0.2 ~ 0.8V in the present work.

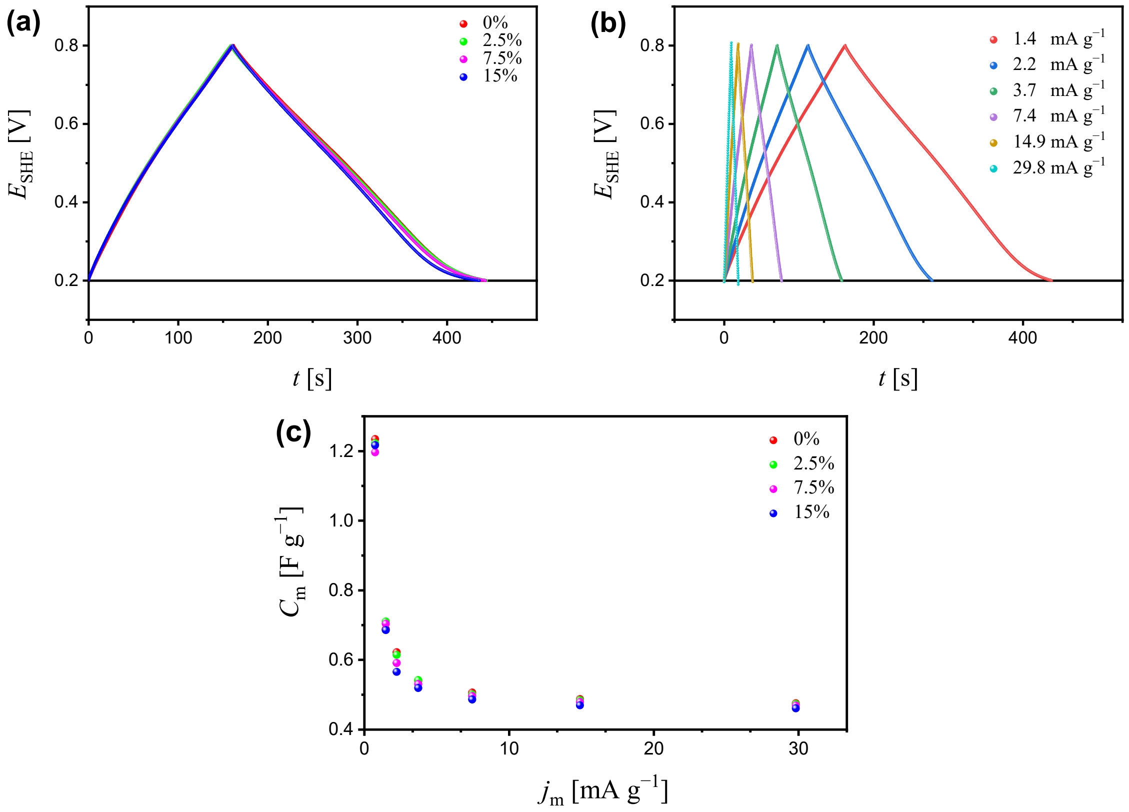

Figure S5. (**a**) Galvanostatic charge-discharge (GCD) curves of NPG at current density j_m = 1.4 mA g^{-1} under various deformations. (**b**) GCD curves of NPG at j_m = 1.4 ~ 29.8 mA g^{-1} under zero deformation. (**c**) Specific capacitance, C_m, as a function of j_m based on the GCD results of NPG.

Figure S5a illustrates the galvanostatic charge-discharge (GCD) curves of NPG at current density j_m = 1.4 mA g^{-1} under various deformations. The charging curves overlap under all deformations, while the discharging time, especially at the deformation of 15%, decreases. Moreover, linear and symmetric charging-discharging curves are also obtained at various current densities, as shown in Figure S5b. In all cases, no evident potential drop is observed even at the largest deformation and discharging j_m. According to the GCD curves, specific capacitance, C_m, under varied deformations are evaluated and plotted versus j_m, as displayed in Figure S5c. Clearly, C_m under all deformations decreases with j_m until j_m = 7.4 mA g^{-1}, beyond which, C_m remains constant. It is noted that at each j_m, a reduced C_m is obtained under deformation.

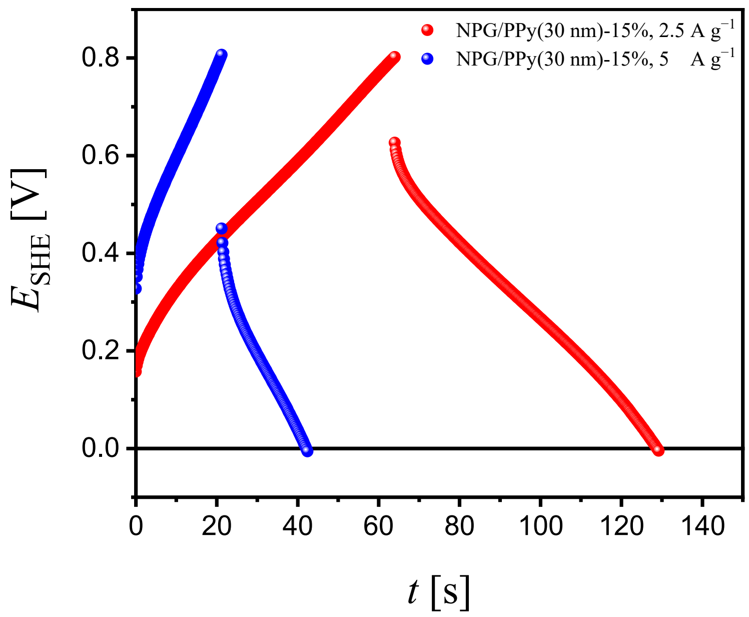

Figure S6. Representative GCD curves of NPG/PPy (30 nm) at current densities of 2.5 and 5 A g^{-1} under a deformation of 15%.

Figure S6 displays typical GCD curves of NPG/PPy (30 nm) at current densities of 2.5 and 5 A g^{-1} under a deformation of 15%. In contrast to 2.5 A g^{-1}, a larger potential drop and faster discharging are observed at 5 A g^{-1}. Yet, the discharging time, discharging current, and discharge potential in Equation 2 synergistically lead to higher specific capacitance at 5 A g^{-1}, as shown in Figure 3d in the main text.

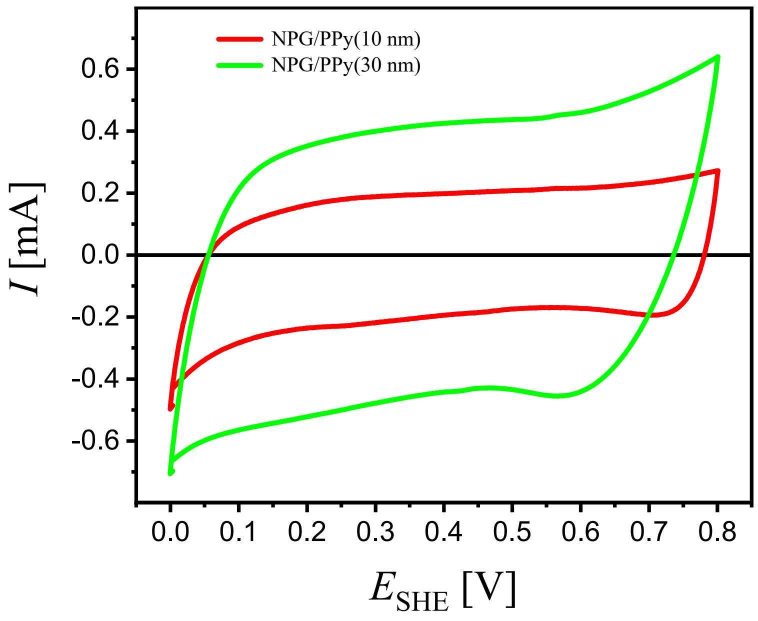

Figure S7. CV curves of NPG/PPy (10 nm) and NPG/PPy (30 nm) recorded at 5 mVs^{-1} in 0.1 M $HClO_4$.

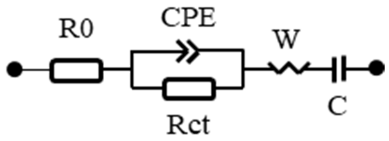

Figure S8. Equivalent circuit for EIS fitting. R_s, bulk electrolyte resistance and any contact resistance; R_{ct}, charge transfer resistance; W, Warburg impedance corresponding to diffusion process; *C*, capacitance induced from surface adsorption; CPE, Constant Phase Element related to the diffusion process.
